# Dexamethasone Promotes a Stem-Like Phenotype in Human Melanoma Cells *via* Tryptophan 2,3 Dioxygenase

**DOI:** 10.3389/fphar.2022.911019

**Published:** 2022-06-30

**Authors:** Marta Cecchi, Antonella Mannini, Andrea Lapucci, Angela Silvano, Matteo Lulli, Cristina Luceri, Mario D’Ambrosio, Alberto Chiarugi, Ali H. Eid, Astrid Parenti

**Affiliations:** ^1^ Department of Neuroscience, Psychology, Drug Research and Child Health, Neurofarba, Pharmacology and Toxicology Section, University of Florence, Florence, Italy; ^2^ Department of Experimental and Clinical Medicine, Internal Medicine Section, University of Florence, Florence, Italy; ^3^ Department of Health Sciences, Clinical Pharmacology and Oncology Section, University of Florence, Florence, Italy; ^4^ Department of Experimental and Clinical Biomedical Sciences “Mario Serio”, University of Florence, Florence, Italy; ^5^ Department of Basic Medical Sciences, Qatar University, QU Health, Doha, Qatar

**Keywords:** melanoma, SK-MEL-28, A375, dexamethasone, glucocorticoids, tryptophan 2,3 dioxygenase, melanospheres, embryonic stem cell markers

## Abstract

In addition to its well-established immunosuppressive actions, tryptophan 2,3-dioxygenase (TDO) appears to elicit direct effects on tumor cell function. Although TDO has been associated with cancer stemness, its involvement in melanoma stem cell biology remains largely unknown. Since we showed that by upregulating TDO, dexamethasone (dex) promotes proliferation and migration of SK-Mel-28 human melanoma cells, we sought to investigate dex effects on melanoma spherogenesis and stemness, and whether these events are mediated by TDO. We demonstrate here that dex significantly upregulates TDO in A375, a more aggressive melanoma cell line, confirming that dex effects are not limited to SK-Mel-28 cells. Moreover, dex stimulates spherogenesis of both cell lines, which is mediated by TDO, evident by its suppression with 680C91, a TDO inhibitor. The formed melanospheres appear to be enriched with embryonic stem cell marker mRNAs, the expression of which is potentiated by dex. Expression of cancer stem cell markers (CD133, CD44, ganglioside GD2) was significantly increased in A375 spheres, as detected by flow cytometry. Taken together, our results suggest that TDO could represent a promising target in the management of melanoma and that dex, routinely used as a co-medication also in advanced melanoma, may stimulate melanoma cell function/tumor-supporting properties, a rather debilitating and undesired side effect.

## Introduction

Degradation of tryptophan (Trp) into kynurenine (kyn) via the kynurenine pathway (KP) is known to play an important role in cancer progression (Opitz et al., 2020). Indeed, conversion of trp to kyn by either tryptophan 2,3-dioxygenase (TDO) or indoleamine 2,3-dioxygenase (IDO1/2) can activate ligand-activated transcription factor aryl hydrocarbon receptor (AhR), which in turn upregulates genes involved in a tolerogenic phenotype (Schramme et al., 2020). While IDO1 has been shown to be primarily involved in immune regulation of cancer, TDO has been largely ignored. TDO is constitutively expressed in several organs including the brain and placenta, as well as the liver where it catalyzes the excess dietary Trp. Recently, it was shown that TDO is expressed in different human tumors, where it sustained kyn production thereby weakening cellular immunity (Schramme et al., 2020).

TDO is constitutively expressed in malignant glioma and triple-negative breast carcinoma, and its upregulation has been associated with immune escape (Opitz et al., 2011; D’Amato et al., 2015). Recently, higher TDO expression was shown to be significantly correlated with immune cell infiltrates and with a worse prognosis in breast cancer patients (Liu et al., 2020). This prompted the notion that TDO may represent an attractive target, especially when IDO1 does not account for constitutive Trp catabolism. While TDO’s role in cancer immunosuppression is well-established (Ye et al., 2019), its direct effects on tumor cells remain unclear. We previously demonstrated that the SK-Mel-28 human melanoma cell line possesses a functional TDO, which is up-regulated by dexamethasone (dex), a synthetic glucocorticoid (GC) widely used in cancer therapy (Cecchi et al., 2021). Dex promoted SK-Mel-28 proliferation, migration, and invasiveness via TDO (Cecchi et al., 2021), highlighting the controversial role of GC in cancer cell fate and the new possible implications of TDO in human melanoma. Melanomas arise from mature, differentiated melanocytes as well as from tumor stem cells that can self-renew and promote incessant growth (Reya et al., 2001). Indeed, a heterogeneous subpopulation of cells, with different phenotypes and stem-cell like properties, contributes to the formation of melanomas (Fang et al., 2005; Zimmerer et al., 2013). Melanoma stem cells, in particular, are characterized by the expression of embryonic transcription factors (Vandamme et al., 2014; Seftor et al., 2019), including NANOG (Homeobox protein), Oct4 (octamer-binding transcription factor 4) and Sox2 (sex-determining region Y HMG-box 2). These proteins are they key factors of self-renewal and tumorigenicity, as well as resistance to anticancer therapies and invasiveness of melanomas, among others (Borrull et al., 2012; Santini et al., 2014).

The use of GCs for the treatment and management of several cancers has been well established, particularly in selected combination therapy regimens. However, the ability of GCs to modulate cancer progression is quite controversial. While GCs are indeed routinely used to induce apoptotic cell death in hematological malignancies (Lin and Wang, 2016) or to alleviate the side effects of treatment (Shih and Jackson, 2007), several lines of evidence implicate GCs as promoters of cancer cell invasion and survival (Herr et al., 2003; Obradović et al., 2019). Discrepant dex effects on tumor cell growth and survival may result from its different effects on specific miRNAs and downstream targets (Mohammadi et al., 2021). MiRNAs have gained more attention in recent years, as they allow the understanding of the regulatory mechanisms of the tumor process. However, the effects of GC on miRNAs responsible for TDO mRNA (TDO2) regulation or melanoma cell function are unknown, highlighting the need to elucidate GC effects on solid tumor cell function and, among them, on melanoma.

Recently, it was reported that dex may decrease the efficacy of chemotherapy by virtue of its ability to induce a stem cell phenotype in ovarian cancer cells, thus promoting cancer progression (Karvonen et al., 2020). In this context, we recently reported that the proliferative effects of dex on the SK-Mel-28 melanoma cell line were mediated by TDO activation. Intriguingly, new evidence alluded to the notion that TDO may play an important role in cancer stem cells (CSCs) (Pham et al., 2018). Indeed, expression of TDO in esophageal CSC (ESCCs) correlates with the expression of the stem marker CD44 (Pham et al., 2018). However, whether TDO or KP are involved in melanoma stemness remains lacking. Here, we investigate the relationship between dex, TDO, and melanoma stemness in SK-Mel-28 and A375 melanoma stem-like subsets and in their parental cells. Our results propose a new insight into the potential contribution of dex and TDO to increased stemness of melanoma cancer, and the consequent implications of the use of dex in the management of melanomas.

## Material and Methods

### Cell Culture

SK-Mel-28 and A375 (ATCC, Manassas, VA, United States), human metastatic melanoma cell lines, were grown in high d-glucose DMEM, with 10% (v/v) heat inactivated fetal bovine serum (HyCloneTM Defined FBS; ThermoFisher Scientific, Waltman, MA, United States), 100 U/mL penicillin, 100 μg/ml streptomycin and 2 mmol/L glutamine (Euroclone S.p.A. (Pero, Milan, Italy) in a humidified chamber with 5% CO_2_ in air. The culture medium was changed every 2 days.

### Spherogenesis and Sphere-forming Efficiency

Melanospheres were generated from A375 and SK-Mel-28 human melanoma cells as previously reported with few modifications (Mannini et al., 2020). Cell culture plates were coated with poly-2-hydroxyethyl methacrylate (poly-HEMA) (Sigma Aldrich, St. Louis, MO, United States) for low attachment conditions. Cells were seeded (12000 viable cells/well (3125 cells x cm^2^) for SK-Mel-28 and 7600/well (2000 cells x cm^2^) for A375 in selective serum-free Dulbecco’s modified Eagle’s medium (DMEM)/Ham’s F12 medium supplemented with 1X B27 supplement (Life Technologies, Carlsbad, CA, United States), human recombinant epidermal growth factor (4 pg/ml, hrEGF) and basic fibroblast growth factor (240 pg/ml, bFGF) (R&D Systems, Minneapolis, MN, United States). The number of melanospheres was followed up to 5 days, and the sphere-forming efficiency (SFE) was calculated after 5 days as percentage (%) of spheres formed, by counting the number of spheres in the entire well using an inverted microscope with a 10x objective and dividing them by the total number of seeded cells and multiplied by 100.

### Flow Cytometry

Parental melanoma cells and melanospheres were labelled and analyzed by direct immunofluorescence staining with a panel of monoclonal antibodies: CD133-phycoerythrin (PE) (Miltenyi Biotec, Bergisch Gladbach, Germany), CD44-allophycocyanin (APC), (eBioscience, San Diego, CA, United States), anti-hGD2 (purified mouse anti-human disialoganglioside GD2, Clone 14.G2a) (BD Pharmingen, Franklin Lakes, NJ, United States). Isotype-matched antibodies were used as negative control and dead cells were excluded by 7-amino-actinomycin D (7-AAD; Sigma-Aldrich) (Paccosi et al., 2014). Results are expressed as percent positive cells.

### RNA Extraction and Quantitative Real Time PCR

Total RNA was isolated from SK-Mel-28 and A375 melanoma cell lines by means of NucleoSpin Total RNA kit according to the manufacturer’s protocol (Macherey-Nagel, Allentown, PA, United States). RNA was quantified by Qubit™ 3.0 Fluorometer (Invitrogen, Waltham, MA, United States). After quantification, total RNA was analysed to determine the quality of samples by the Agilent RNA 6000 Nano LabChip^®^ kit with the Agilent 2100 Bioanalyzer (Agilent Technologies, Santa Clara, CA, United States). Two hundred ng of total RNA from samples obtained as described above were retro-transcribed using iScript (Bio-Rad, Hercules, CA, United States) in 20 µL of total volume, and amplified with specific primers listed in Table 1, in 20 µL. PCR amplification was carried out by SsoAdvancedTM universal SYBR^®^ Green Supermix (Bio-Rad, United States) according to manufacturer’s instructions using a RotorGene 3000 (Qiagen, Hilden, Germany) instrument. In the present analysis, 18s rRNA was confirmed to be stable and was used as the internal control. RT-qPCR was performed using the following procedure: 98°C for 2 min, 40 cycles of 98°C for 5 s, 60°C for 10 s. The program was set to reveal the melting curve of each amplicon from 60 to 95°C with a read every 0.5°C.

**TABLE 1:** Sequences of primers used for qRT-PCR.

Genes	Forward	Reverse
*TDO2*	CTT​ATC​TCC​AGC​ATC​AGG​CTT​CCA​GAG​T	GGA​GTT​CTT​TCC​AGC​CAT​GCC​TCC
*IDO1*	AGT​TCT​GGG​ATG​CAT​CAC​CA	CAG​TTT​CTT​GGA​GAG​TTG​GCA
*AHR*	CAA​ATC​CTT​CCA​AGC​GGC​ATA	CGC​TGA​GCC​TAA​GAA​CTG​AAA
*E2F1*	GAG​ACC​TCA​CTG​AAT​CTG​ACC​ACC​AA	CCA​GTT​CAG​GTC​GAC​GAC​ACC​GT
*OCT-3/4*	CCT​GGA​GAA​TTT​GTT​CCT​GCA​GTG​CC	CTC​GAG​CCC​AAG​CTG​CTG​GGC​G
*SOX2*	CCC​CTG​GCA​TGG​CTC​TTG​GCT​CC	GGA​AGA​GGT​AAC​CAC​AGG​GGG​GC
*c-MYC*	GAC​GAG​GAG​GAG​AAC​TTC​TAC​CAG​C	TTT​CTT​CCA​GAT​ATC​CTC​GCT​GGG​CG
*KLF4*	TCA​GCG​ACG​CGC​TGC​TCC​CAT​CTT​T	CAC​CTG​CTT​GAC​GCA​GTG​TCT​TCT​C
*NANOG/P8*	GCC​TGG​AAC​AGT​CCC​TTC​TAT​AAC​TG	CTG​GCA​GGA​GAA​TTT​GGC​TGG​AAC​TG
*AP-1/JUN*	TGG​AAA​CGA​CCT​TCT​ATG​ACG​ATG​CCC	TTG​GGG​TTA​CTG​TAG​CCA​TAA​GGT​CCG
*18s rRNA*	CGG​CTA​CCA​CAT​CCA​AGG​AA	GTT​ATT​TTT​CGT​CAC​TAC​CTC​CCC​GGG

### miRNA-specific cDNA Synthesis

For miRNA-specific cDNA synthesis, total RNA was reverse transcribed by using the miRCURY LNA RT kit (Qiagen). Quantitative real-time PCR amplification and analysis were performed using the Rotor-Gene Q thermal cycler (Qiagen), the miRCURY LNA SYBR^®^ Green PCR kit and specific miRCURY LNA miRNA PCR Assay for miR-200c and miR-155-3p. The cycle was set at 9°C for 15 s, 55°C for 30 s and 70°C for 30 s, repeated 40 times. RNU-6B was used as an endogenous control. Relative expressions between miRNAs and RNU-6B were calculated by using a 2^−ΔΔCt^ formula.

### Immunofluorescence

A375 were seeded (1.5 × 10^4^ cells) in high d-glucose DMEM with 10% FBS into LabTek Slides Chamber and incubated in a 5% CO_2_ atmosphere at 37°C for 24 h. Fresh medium with low serum concentration (1% FBS) was then added, and cells were stimulated with dex every 3 h up to 12 h. Double immunofluorescence analyses were performed on cells after fixation with cold acetone for 5 min. Non-specific binding sites were blocked with 10 mg/ml bovine serum albumin in PBS for 1 h at room temperature, with 0.2% triton X-100 (Sigma-Aldrich). A primary antibody (a monoclonal rabbit anti-human AhR (1: 100; Cell Signaling Technology, Danvers, MA, United States), polyclonal rabbit anti-human IDO1 (1:200; Abcam, Cambridge, United Kingdom) or monoclonal anti human-TDO (1:200; Novus Biologicals, Briarwood Ave, OH, United States)) was then added overnight at 4°C, followed by washing. Then a 2 h incubation at room temperature with a secondary goat anti-rabbit or anti-mouse antibodies, conjugated with FITC AF488 (green fluorescence) (all from Life Technology). The signal was amplified with anti-FITC Fluorescein/Oregon green antibody for 1 h 30 min (1:100; Invitrogen) at room temperature. Nuclei were labelled with Hoechst 33342 (20 μg/ml; Sigma-Aldrich; blue fluorescence). Omission of primary antibodies was used as negative controls. Slides were mounted with Fluoromount (Sigma-Aldrich, MO, United States) and examined with Leica DC200 microscope digital color camera and Leica DC Viewer software.

### Statistical Analysis

Statistical analysis was conducted using GraphPad Prism 8.00 (GraphPad Software, San Diego, CA, United States). Parametric data were reported as means ± SEM and differences between groups were tested with ANOVA test (followed by Bonferroni’s and Dunnett’s Multiple Comparison Test). For comparisons between two groups, we used Student’s unpaired *t* test. Alpha value was set at 0.05.

## Results

### Effect of Dexamethasone on TDO, IDO1 and AhR Expression in A375 Melanoma Cells

**FIGURE 1:**
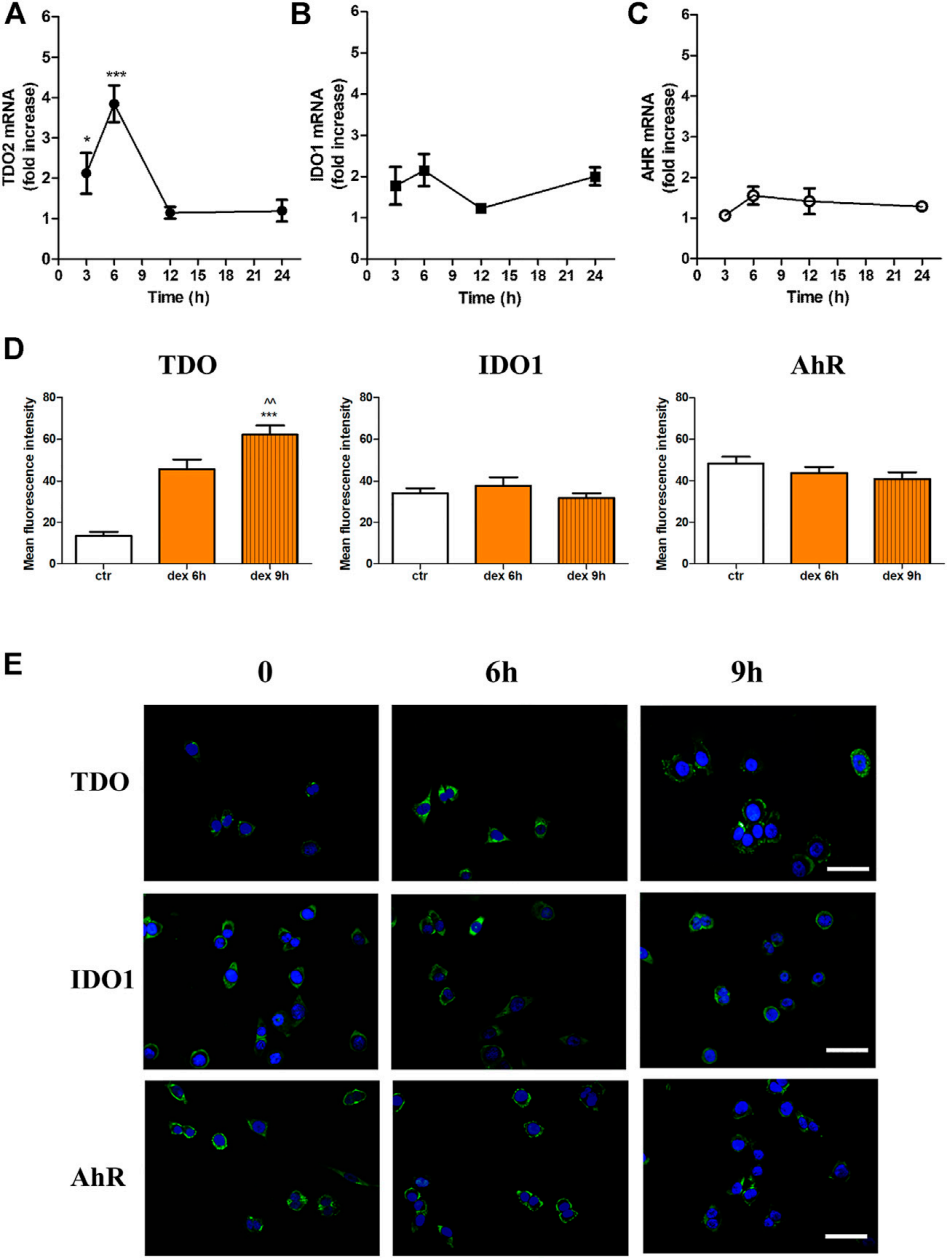
Effect of Dex on TDO **(A)**, IDO1 **(B)**, and AhR **(C)** mRNA of A375 melanoma cells. Cells were treated with dex (25 µM) for different time-points. Real-time RT-PCR shows time-dependent increase in mRNA of TDO2. Data are plotted as mean ± SEM (n = 8, * denotes *p* < 0.05, and ***p < 0.001 vs. control unstimulated cells) **(D)** Mean fluorescence intensity of TDO, IDO1 and AhR expression in control unstimulated cells (ctr) and cells stimulated with dex for 6 and 9 h (n = 3; *** denotes *p* < 0.001vs. control unstimulated cells, and ^^ *p* < 0.01 vs. dex 6h **(E)** Immunofluorescence for TDO, AhR and IDO1 expression. Cells were treated without (0), or with dex (25 µM) for 6 and 9 h. Representative photomicrographs at 40X magnification are shown. Scale bar 20 µm.

We recently showed that TDO is expressed in SK-Mel-28 cells, and that this expression is upregulated by dex with maximal effects achieved at 25 µM (Cecchi et al., 2021). To test whether this expression is restricted to this cell line, we employed a more aggressive melanoma cell line, namely A375 (Rossi et al., 2018). We sought to determine the expression of TDO, IDO1 and one of their downstream effectors, the aryl hydrocarbon receptor (AhR), which we previously reported to be upregulated in SK-Mel-28. A375 cells were stimulated with dex for increasing time durations, and mRNA and proteins levels of TDO, IDO1 and AhR were assessed. Our results show that dex significantly upregulated TDO mRNA (TDO2) in a concentration- (data not shown) and time-dependent manner, with maximal effect after a 6 h stimulation with 25 μM, where a 3.95 ± 0.42-fold increase was noted (*p* < 0.001) (Figure 1A). Conversely, dex did not significantly affect either IDO1 or AhR mRNAs (Figures 1B,C).

This increase in mRNA expression was concomitant with increased protein expression. Quantitative analysis showed significant increase in the immunofluorescent signal of TDO but not AhR or IDO1 (Figure 1D). Dex-induced upregulation of TDO starts after 6 h, maximal expression is achieved after 9 h (Figure 1D) and then declines to basal levels within 24 h (data not shown). Representative photomicrographs for IDO1 or AhR evident by lack of an appreciable change in immunofluorescent studies (Figure 1E).

### Effects of Dex on miRNAs

**FIGURE 2:**
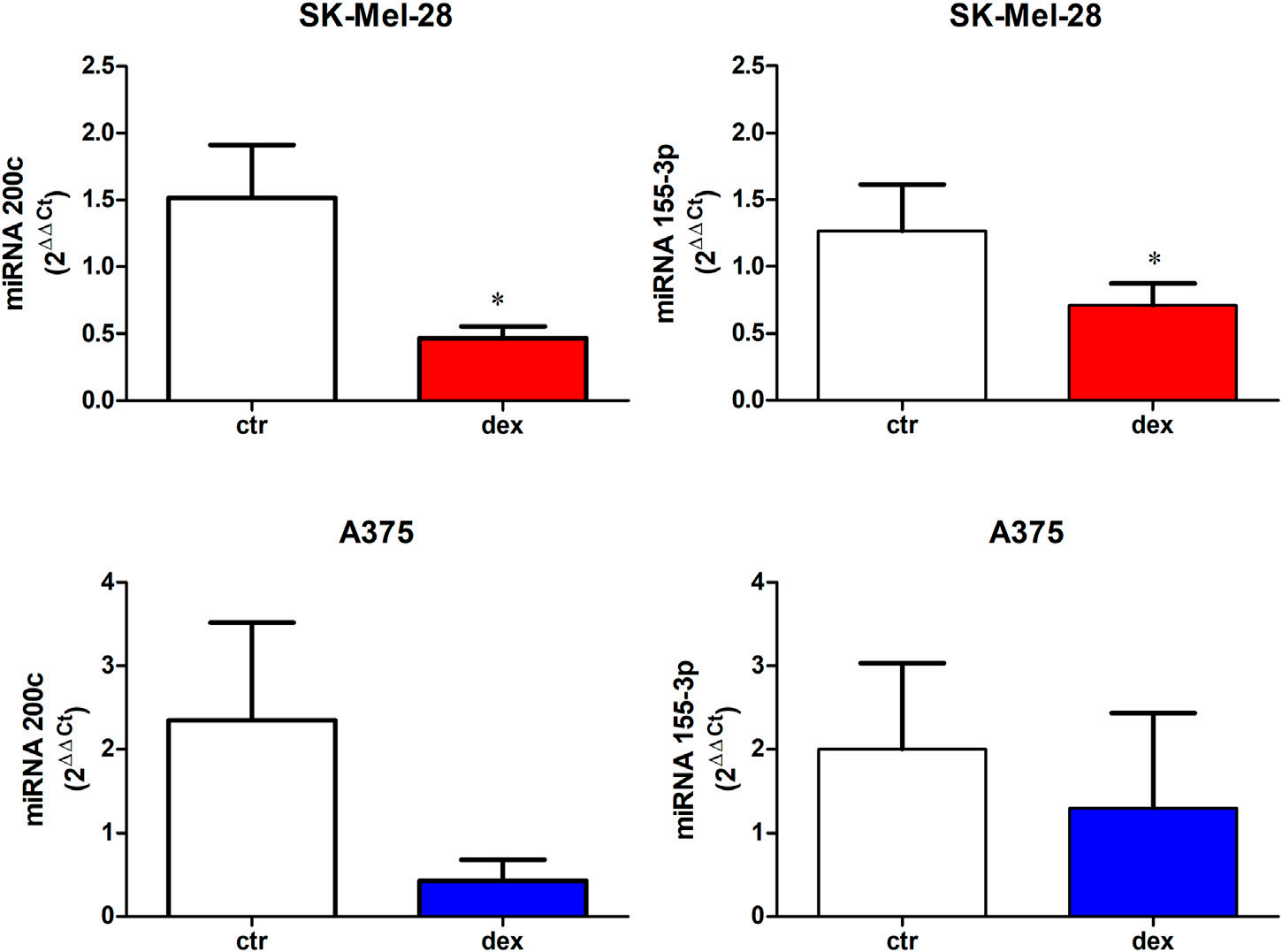
miRNA 200c and miRNA 155-3p expression in untreated (ctr) or dex-treated SK-Mel-28 cells and A375 cells. Cells were treated with dex (25 µM) for 6 h; total cDNA was then extracted, and RT-PCR was performed. Data represent mean ± SE, n = 4. * denotes *p* < 0.005.

Melanoma progression is associated with the expression profile of miRNA-200 family (Van Kempen et al., 2012). Interestingly, TDO expression is known to be modulated by miRNA-200c (Rogers et al., 2019). Moreover, recent evidence suggests that the miRNA-155 family, which is expressed in melanomas, is correlated with TDO expression and clinical outcome in kidney renal carcinoma (Yang et al., 2020). We then investigated whether dex affects the expression of miRNA-200c and miRNA-155-3p. Figure 2 shows that treatment with dex significantly downregulated both miRNAs in SK-Mel-28, inducing a 66 ± 9.5 and 38 ± 8.2 inhibition for miRNA200c and miRNA-155 expression, respectively. Dex inhibited both miRNAs in A375 cells, albeit not statistically significant.

### Dexamethasone Stimulates Spherogenesis and Increases Cancer Stem Cell Markers

**FIGURE 3:**
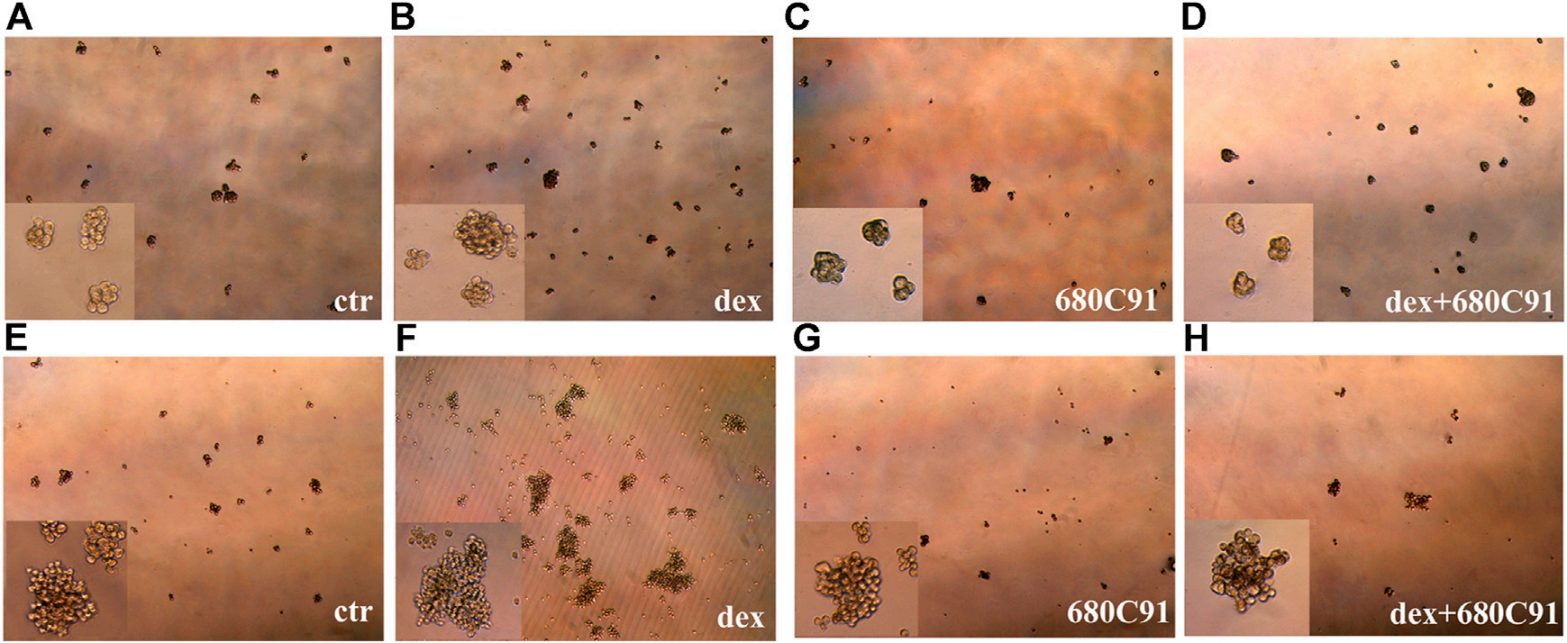
SK-Mel-28 **(A-D)** and A375 **(E-H)** melanospheres on day 5 of culture. SK-Mel-28 and A375 parental cells were untreated (ctr) or treated with dex or/and TDO selective inhibitor 680C91 for 24 h and then seeded into low-attachment conditions. SFA was followed every day for up to 5 days. Photomicrographs of the melanospheres were taken, and representative images are shown (total magnification 40x). Insert pictures: total magnification 100x.

Cancer stem cells (CSCs) are pivotal for cancer initiation and recurrence, and several studies have demonstrated that melanoma stem cells are crucial for both initiation of the disease and metastasis. Since sphere formation assay (SFA) is the gold standard assay to evaluate self-renewal and differentiation of stem cells at the single-cell level *in vitro* (Tuccitto et al., 2017), we investigated the effect of dex on SFA of both SK-Mel-28 and A375 cells, and whether TDO inhibition modulates this potential spherogenesis. Both cell lines were able to form melanospheres, with the kinetics of A375-formed spheroids being faster than SK-Mel-28’s (data not shown). A complex and heterogeneous morphology was reached within 5 days. SK-Mel-28 formed small compact spheroid masses of cells (Figure 3A–D), while A375 cells form spheres with more complex morphology (Figure 3E–H).

**FIGURE 4:**
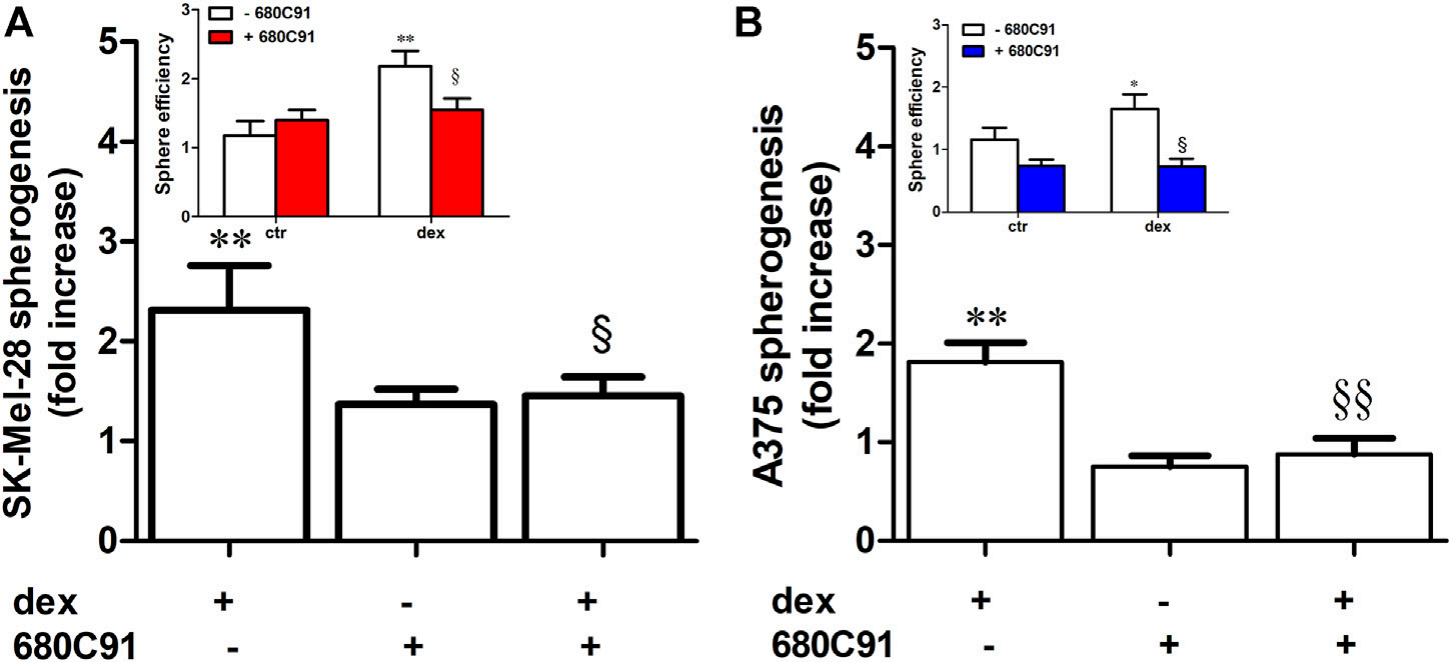
Dexamethasone stimulates spherogenesis via TDO. Adherent SK-Mel-28 **(A)** or A375 **(B)** were stimulated with dex (25 µM) for 24 h in the presence or absence of 680C91. Cells were collected and then seeded under low-attachment conditions. Sphere number was assessed after 5 days. Data are shown as mean ± SE of sphere efficiency and as fold increase in spherogenesis. **p < 0.01 vs. unstimulated cells; § denotes *p* < 0.05, §§ denotes *p* < 0.01 vs. dex alone; n = 5.

Dex significantly increased the number of spheres of both A375 and SK-Mel-28, with this effect being more pronounced in SK-Mel-28 (Figure 4). The number of spheres with dimensions more than 225 × 225 µm in A375 were significantly increased by dex as well, and this effect was prevented by 680C91. Indeed, the number of spheres reaching that dimension was 8.25 ± 0.8, 14.2 ± 2.5, and 9.75 ± 0.8 in control unstimulated, dex-stimulated, dex-stimulated and 680C91-pretreated cells, respectively (*p* < 0.05). Interestingly, TDO inhibition did not affect sphere formation of unstimulated cells, but it significantly abolished the effect of dex in both melanoma cell lines (Figures 3, 4). Indeed, in SK-Mel-28 cells, there was a 1.3 ± 0,7 and 2.31 ± 0.3-fold increase in spheres in the presence of absence of 680C91, the TDO inhibitor (Figure 4). Similarly, in A375 cells, there was a 0.87 ± 0.16 and 1.81 ± 0.19-fold increase in the presence of absence of 680C91 (Figure 4).

**FIGURE 5:**
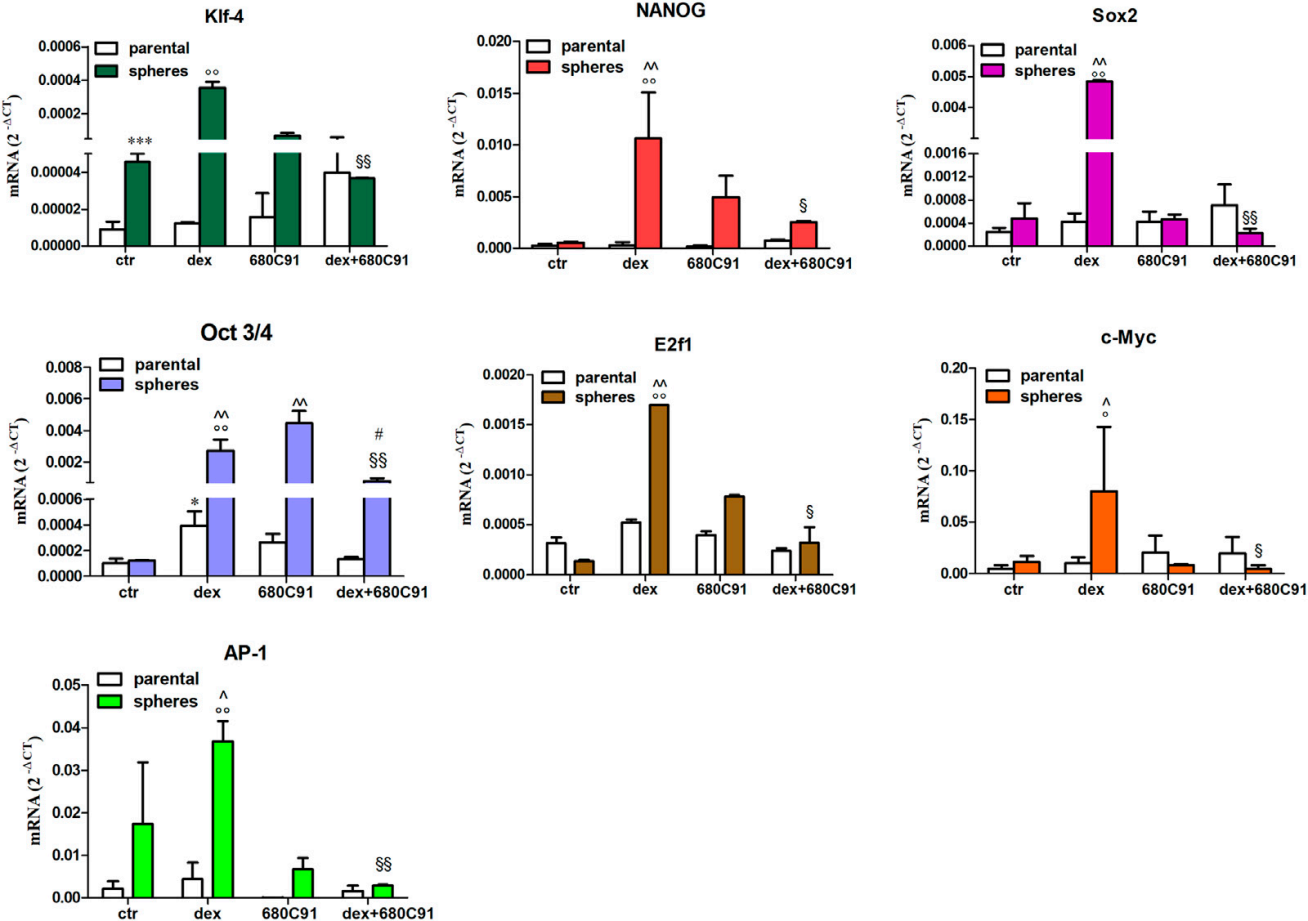
Relative expression level of stemness marker mRNAs expression in SK-Mel-28 spheres. Parental cells in culture were treated for 24h with dex (25 µM) in the absence or presence of 680C91 (40 µM). Cells were then detached and seeded in low-attachment dishes to induce sphere formations. After 5 days, total RNA was extracted, and real-time PCR was performed. Data are plotted as mean ± SE, n = 3. *p < 0.05; ***p < 0.001 vs. unstimulated (ctr) parental; °*p* < 0.001 vs. dex parental; ^*p* < 0.001 vs. unstimulated (ctr) spheres; § denotes *p* < 0.05, §§ denotes *p* < 0.01 vs. dex spheres; # denotes *p* < 0.01 vs. 680C91-spheres.

To demonstrate the enrichment of CSC characteristics in the spheres, expression of pluripotent embryonic stem cell markers in both lines was analyzed and compared with that expressed by parental cells. To this end, we first sought to assess whether dex modulates the expression of those markers in parental cells, and whether TDO is involved in this dex-mediated expression. Dex significantly increased the expression of Oct3/4, and AP-1, E2f1, albeit with a less effect, in SK-Mel-28. This up-regulation was inhibited by the TDO inhibitor 680C91 (40 µM) (Figure 5). In unstimulated spheres, there was no change in the expression of Oct3/4 (1.43 ± 0.3), c-Myc (1.3 ± 0.4), Sox2 (2.66 ± 1.9-fold), E2f1 (0.5 ± 0.3-fold), an increase of NANOG (5.55 ± 2.2-fold), AP-1 (80.4 ± 28-fold) (*p* > 0.05), and a significant increase of Klf4 (51.3 ± 25, *p* < 0.05) compared to parental cells. However, spheres derived from parental cells that had been stimulated with dex exhibited a higher increase in all stem cell markers. Indeed, the mean fold increase was 7.18 ± 3.4, 16.4 ± 6.1, 7.25 ± 4.0, 7.06 ± 2.1, 13.91 ± 7.7, 22.8 ± 5.6 for Ap-1, NANOG, Klf4, c-Myc, Sox2, Oct3/4, E2f1, respectively (Figure 5). Importantly, TDO inhibition significantly abrogated the dex-induced expression of these markers (Figure 5). Interestingly, expression of Oct3/4 in spheres obtained from 680C91-treated parental cells was upregulated compared to that of control. However, its expression in spheres derived from dex +680C91-treated parental cells was significantly reduced, at lower levels than those induced by 680C91 alone.

**FIGURE 6:**
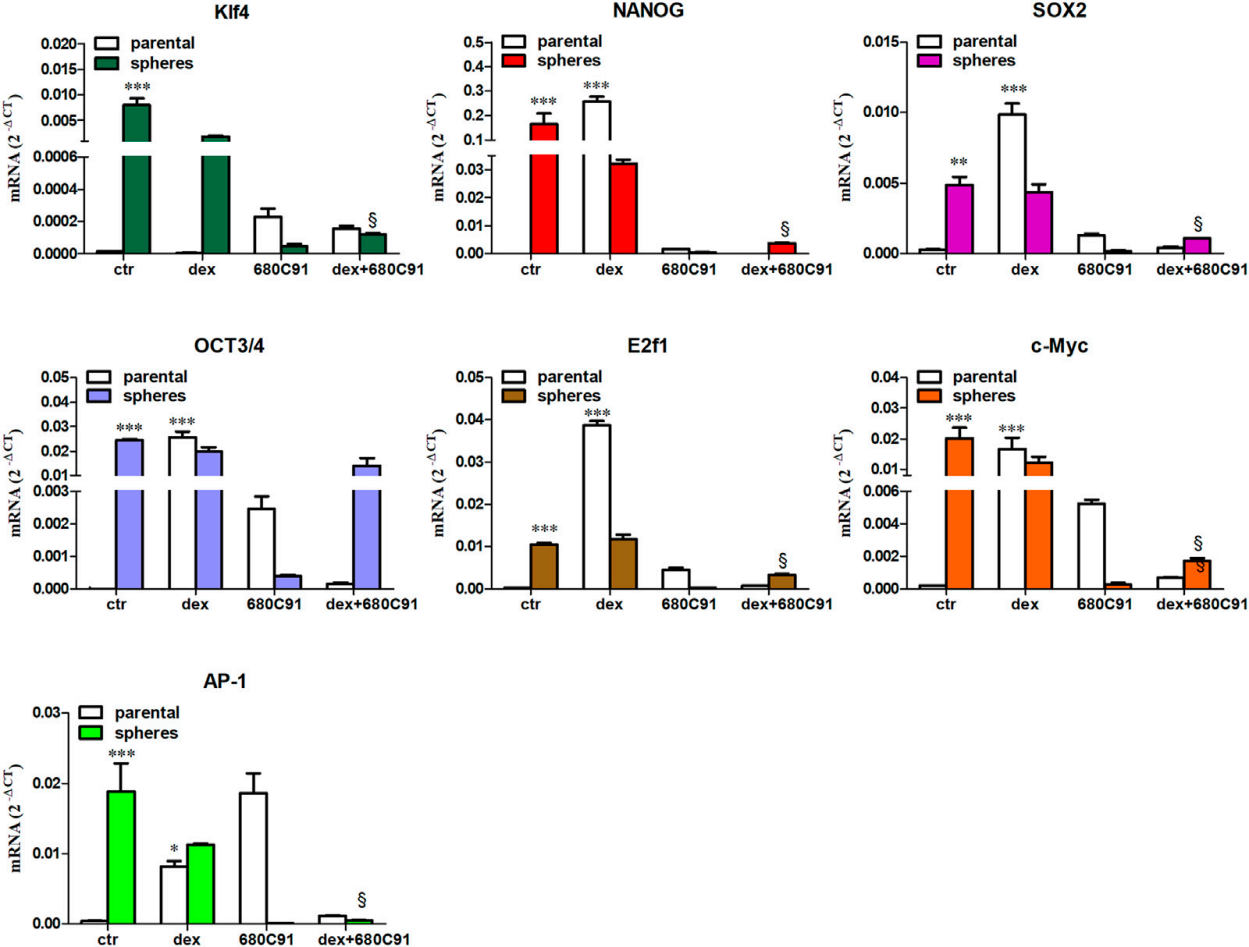
Relative expression level of stemness marker mRNAs expression in A375 spheres. Parental cells in culture were stimulated with dex ±680C91 for 24 h. Then cells were detached and seeded in low-attachment dishes to induce sphere formations. After 5 days, total RNA was extracted to perform real-time PCR. Data are shown as Mean ± SE, n = 3. ** denotes *p* < 0.01, *** denotes *p* < 0.001 vs. unstimulated (ctr) parental; §§*p* < 0.01 vs. dex spheres.

In parental A375, basal expression of stem cell markers mRNAs was relatively undetectable. However, treatment with dex causes a significant increase in all stem cell markers, except Klf4. Indeed, there was a 19.6 ± 1.7, 75376 ± 884, 84.07 ± 2.6, 33.76 ± 2.6, 2505 ± 242, and 170.2 ± 5.31-fold increase in AP-1, NANOG, c-Myc, Sox2, Oct3/4 and E2f1, respectively (Figure 6). Like in SK-Mel-28 cells, dex-induced expression was abolished by 680C91 (Figure 6). In A375-derived melanospheres, expression of stem cell markers was significantly higher in control unstimulated cells compared to parental ones. Indeed, a 563.2 ± 92, 48608.7 ± 12000, 16.6 ± 2,8, 2401.2 ± 58, 46.01 ± 1,99, 101.8 ± 17,4, and 45.06 ± 9,54-fold expression for Klf4, NANOG, Sox2, Oct3/4, E2f1, c-Myc and AP-1, respectively, was noted (Figure 6). Treatment with dex did not augment the increase further, which could be due to the notion that the increased expression was already near maximal or around the plateau value. Consistent with results in the other cell line, treatment of parental cells with 680C91 significantly abolished expression of these stemness markers in control unstimulated spheres.

**FIGURE 7:**
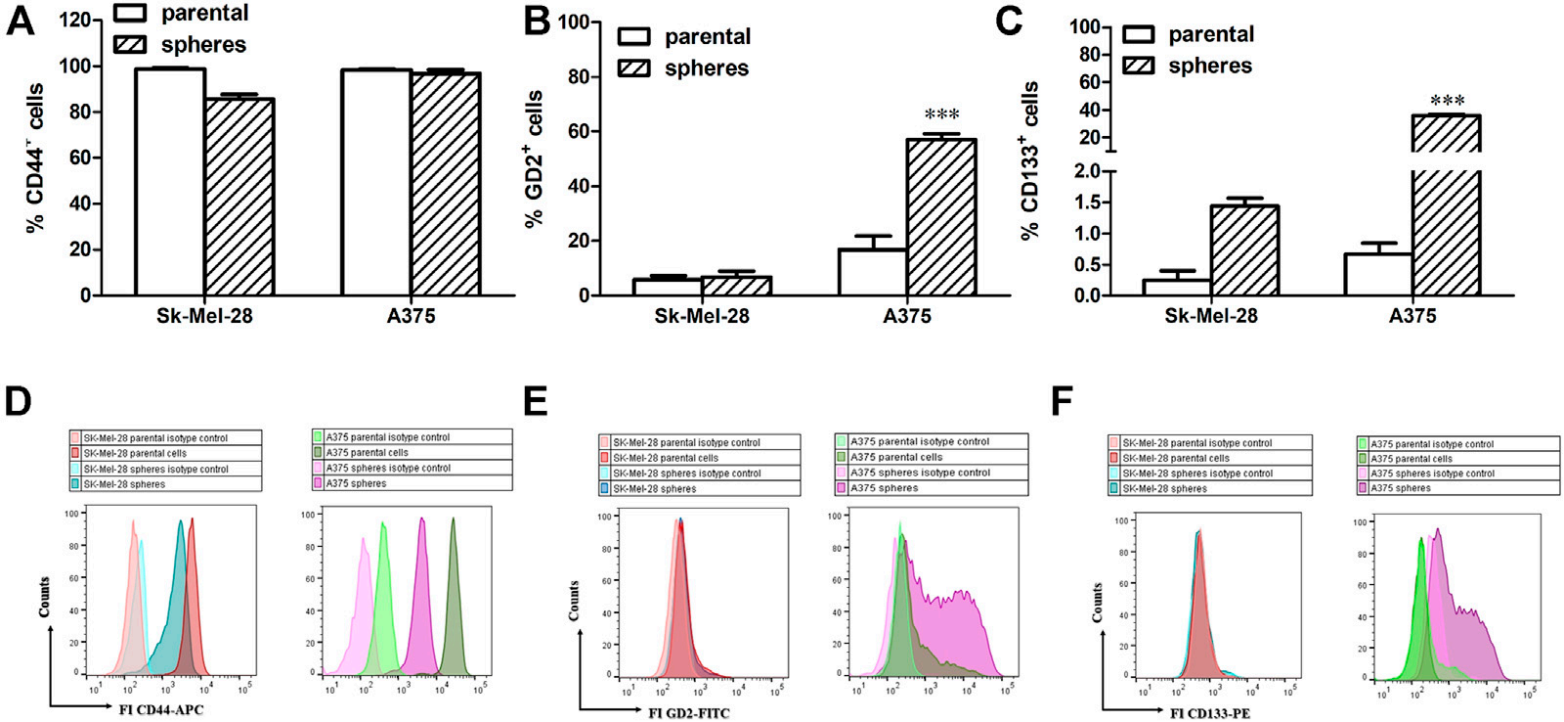
Expression of CD44 **(A,D),** GD2 **(B,E)** and CD133 **(C,F)**. Parental melanoma cells and melanospheres were labelled and analysed by direct immunofluorescence staining with a panel of monoclonal antibodies: hCD133-phycoerythrin (PE), hCD44-allophycocyanin (APC), hGD2. Data are shown as Mean ± SE, n = 4. ** denotes *p* < 0.01, *** denotes *p* < 0.001 vs. parental. **(D–F)** Representative histograms of parental cells and melanospheres in the absence and in the presence of antibodies are shown.

It has been reported that the presence of CD133 or the ganglioside GD2 is associated with increased stemness in many cancers (Monzani et al., 2007; Fan et al., 2010; Battula et al., 2012). However, the role of these two factors in melanoma stemness remains largely obscure. Previous results report that in both parental cell lines, more than 90% of the population exhibit a remarkable expression of CD44 (Mirkina et al., 2014). Our results here show that both SK-Mel-28 and A375 cells exhibit a strong CD44 fluorescence under basal conditions (Figure 7A). Ganglioside GD2 was detectable in a restricted subpopulation in both melanoma cell lines, with higher GD2 positivity rates for A375 than for SK-Mel-28 (Figure 7B). CD133 was scarcely expressed, although more evident in the parental A375 cells, as expected (Figure 7C).

After 5 days in low attachment and serum-free medium conditions, melanoma stem-like subsets (i.e., spheres) showed high levels of CD44 expression, similar to that of the parental cells (Figure 7A). While it showed only a marginal increase in SK-Mel-28 spheres, GD2 expression was significantly increased in A375 spheres (Figure 7B). On the other hand, expression of CD133 on melanoma stem-like subsets was significantly higher in both cell lines. Interestingly, treatment with dex did not modify these CSC patterns in both parental and spheres (data not shown), at least within the 5 days during the spherogenesis process.

## Discussion

It was not until recently that the role of TDO in cancer was appreciated, when emerging evidence showed that TDO, but not IDO1, is the central Trp-degrading enzyme in human glioma cells, and that the TDO–AhR pathway was associated with malignant progression and poor survival for this tumor (Opitz et al., 2011). Owing to its involvement in tumor immune escape, TDO was indeed considered a potential pharmacological target. Recently, experimental evidence suggested a direct effect of TDO on tumor cell survival and proliferation. Moreover, we and others have shown that it promotes growth, migration, and invasiveness of cancer cells, including melanomas (Pham QT. et al., 2021; Cecchi et al., 2021).

More attention has been given to TDO’s role in tumor progression as a result of the interesting finding that glucocorticoids (GCs) increase its expression. GCs, particularly dexamethasone, are routinely used to induce cancer cell death, or to mitigate the undesirable side effects of chemotherapy (Navari and Aapro, 2016). It was also speculated that GCs may suppress tumor growth, although effects of dex on growth of solid tumors remain inconclusive (Aldaz et al., 2021). The clinical relevance of GCs and their effects on the malignant phenotype warrant further investigations.

We previously demonstrated that dex stimulates SK-Mel-28 growth and migration via TDO upregulation. Here, we wished to investigate the possible involvement of dex in spherogenesis and stemness of SK-Mel-28, and the more aggressive A375 melanoma cell line. We also sought to determine whether TDO plays a role in dex-modulated spherogenesis. This becomes increasingly important since cancer stem subpopulations play critical roles in malignancy, making it imperative to better understand the role of dex in melanoma.

Here, we showed that dex upregulates mRNA and protein levels of TDO in A375 cells, demonstrating that dex effects are not only restricted to SK-Mel-28 cells. Furthermore, this dex-mediated effect appears to involve repression of miRNA-200c, a player known to target TDO2 in breast carcinoma cells, resulting in reduced production of the immune-suppressive metabolite kynurenine (Rogers et al., 2019). By suppressing miRNA200c, dex promotes TDO expression and function in melanoma cells. Our data also show that dex suppresses miRNA-155p, a melanoma cell-secreted exosomal miRNA which regulates other miRNAs half-life. Interestingly, reduced miRNA-155 levels are associated with survival of melanoma, while its increased expression significantly reduces SK-Mel-28 invasiveness (Gao et al., 2021).

Our present data could suggest a possible connection between dex treatment and melanoma cell survival or aggressiveness. The pharmacology of glucocorticoids is rather complex and is the subject of ongoing studies that seek to better understand their role in tumorigenesis. Apparently, glucocorticoids may control cell proliferation, migration, and survival by influencing miRNA expression. Unfortunately, however, the effects of dex on cancer cell treatment are still controversial, partly because the effects of dex on the expression of certain miRNAs and downstream targets is itself inconsistent (Mohammadi et al., 2021). Thus, further experimentation is warranted to better elucidate the mechanisms and effects of dex on miRNAs regulation, especially as it pertains to the involvement of TDO in melanoma progression.

Cancer stem cells play increasing roles in promoting tumorigenesis, and thus, targeting them may prove essential in the fight against cancer. To this end, we assessed the involvement of dex and TDO in cancer stemness. Our results demonstrate, for the first time, that dex stimulates the formation of melanospheres in a TDO-dependent mechanism. In Pham’s study (Pham Q. T. et al., 2021), TDO was shown to be up regulated in bladder cancer cells, stimulating their growth and invasiveness. Interestingly, TDO was found to be necessary for spheroid formation in these cells, suggesting that it could be a potential marker for targeted therapy in bladder cancer (Pham QT. et al., 2021). Our results further show that melanospheres obtained from SK-Mel-28 exhibit increased expression of CSC markers such as Oct3/4, AP-1, Klf-4. Interestingly, melanospheres derived from dex-stimulated parental (adherent) melanoma cells displayed a remarkable increase in stem cell markers (Oct3/4, AP-1, E2f1, Sox2 and NANOG), and that this increase was TDO-mediated. Intriguingly, TDO inhibition significantly up-regulated Oct3/4 in unstimulated spheres, notwithstanding its role in suppressing dex-induced up-regulation of this very stemness marker. In A375 cells, all tested stem cell markers except Klf4 were dramatically upregulated by dex in parental cells. Moreover, expression of these markers was higher in control of unstimulated spheres compared to parental cells. While the mechanisms underlying this discordance are unclear, they nonetheless highlight the considerable differences in stem cell panel expression in the 2 cell lines. This, in turn, may at least partly contribute to the heightened aggressiveness of A375 compared to SK-Mel-28, as also suggested by other studies (Rossi et al., 2018).

To the best of our knowledge, this is the first report to show that dex can promote melanoma stemness. This underscores the potential involvement of dex in growth, survival and heterogeneity of melanoma cell. Pluripotency genes of stem cells are known to initiate tumorigenesis by promoting a highly proliferative and aggressively metastatic phenotype.

A key player in melanoma is E2f1, a member of the E2f family of transcription factors involved in cell cycle regulation (Tsantoulis and Gorgoulis, 2005). Increased levels of E2f1 are associated with melanomas, while reduced levels precipitate melanoma cell death, strongly suggesting that E2f1 could be a diagnostic melanoma biomarker (Rouaud et al., 2018). More recently, E2Fs were shown to be upregulated in cutaneous melanoma, and their high expression levels were related to lower overall survival rates and disease-free survival (Li et al., 2021).

Oct4, NANOG and Sox2 are transcription factors that work in concert to maintain ESC pluripotency and self-renewal (Boyer et al., 2005; Loh et al., 2006). Oct4 expression in CSCs is associated with a more aggressive tumor phenotype (Gidekel et al., 2003; Mohiuddin et al., 2020), and in melanoma cells, it is a marker of tumor-initiating cells, metastasis, and resistance to anticancer therapies (Wang and Herlyn, 2015). NANOG and Oct4 overexpression is correlated with increase invasiveness of human melanoma cell lines (Borrull et al., 2012) and NANOG up-regulation is associated with worse prognosis and survival in cutaneous melanoma (Silva et al., 2021). Further, Sox2 is known to regulate self-renewal and tumorigenicity of melanoma cells (Santini et al., 2014). Accumulating evidence is emerging that dex could modulate cancer stem cell markers. Indeed, a recent study reported that in ovarian cancer cells, it was observed the development of a stemness phenotype, mediated by dex upregulation of ROR1 (Karvonen et al., 2020). Contextually, dex increases stem cell marker Sox2, Klf4, NANOG and Oct4 in human mesenchymal stem cells modifying their immunomodulatory property (Rawat et al., 2021). Together, these reports highlight the potential role of dex in cancer stemness-mediated cell survival, growth, and metastasis. What is intriguing and peculiar is that dex-induced melanoma stemness is mediated by TDO activity. Moreover, pretreatment of parental cells with 680C91 significantly abolished expression of these stemness markers in control unstimulated spheres, suggesting that TDO may regulate those genes in unstimulated cells as well. Our recently published report and this study clearly show that dex upregulates TDO (Cecchi et al., 2021), whose inhibition abrogated melanoma cell spherogenesis and suppressed the up-regulation of stem cell markers.

A role for CD133 expression in cancer has been suggested. In this study, we showed that CD133 was expressed in less than 1% of parental A375 and SK-Mel-28 cells, as reported in previous studies (Zimmerer et al., 2013). While there was only a modest increase of CD133^+^ fraction in SK-Mel-28 spheres, a significant increase was observed in A375-derived spheres since they represented 35.7% of cell population. This data could highlight the heterogeneity of melanoma cell lines and may account for the more aggressiveness of A375 compared to SK-Mel-28 (Rossi et al., 2018). Indeed, melanoma-specific CD133^+^ isolated cancer stem cells exhibit increased stem cell markers, such as NANOG and Oct3/4, and possess tumor-initiating potential, compared to CD133^-^ cells (Kumar et al., 2016).

Gangioside GD2 is a sialic acid-containing glycosphingolipid, expressed on different types of tumor cells including melanoma cells (Battula et al., 2012; Dobrenkov and Cheung, 2014). GD2 was reported to represent a CSC marker in different tumors, and several studies suggest that cancer stemness expression requires the integrity of the ganglioside biosynthetic pathway (Battula et al., 2012). Although the specific role played by GD2 in cancer stemness is not well clarified, we sought to investigate whether it was up-regulated in melanospheres formation. Our present data demonstrate that in SK-Mel-28 spheres, GD2 positivity is weakly enhanced, while it is significantly upregulated in A375 spheres, suggesting that the particular ganglioside patterns, belonging to these human melanoma lines, could differently participate in cancer stemness phenotype. However, dex did not modify CSC patterns. It could be interesting to evaluate the expression of those markers, after a longer time of culture, as reported elsewhere (Santini et al., 2014).

**FIGURE 8:**
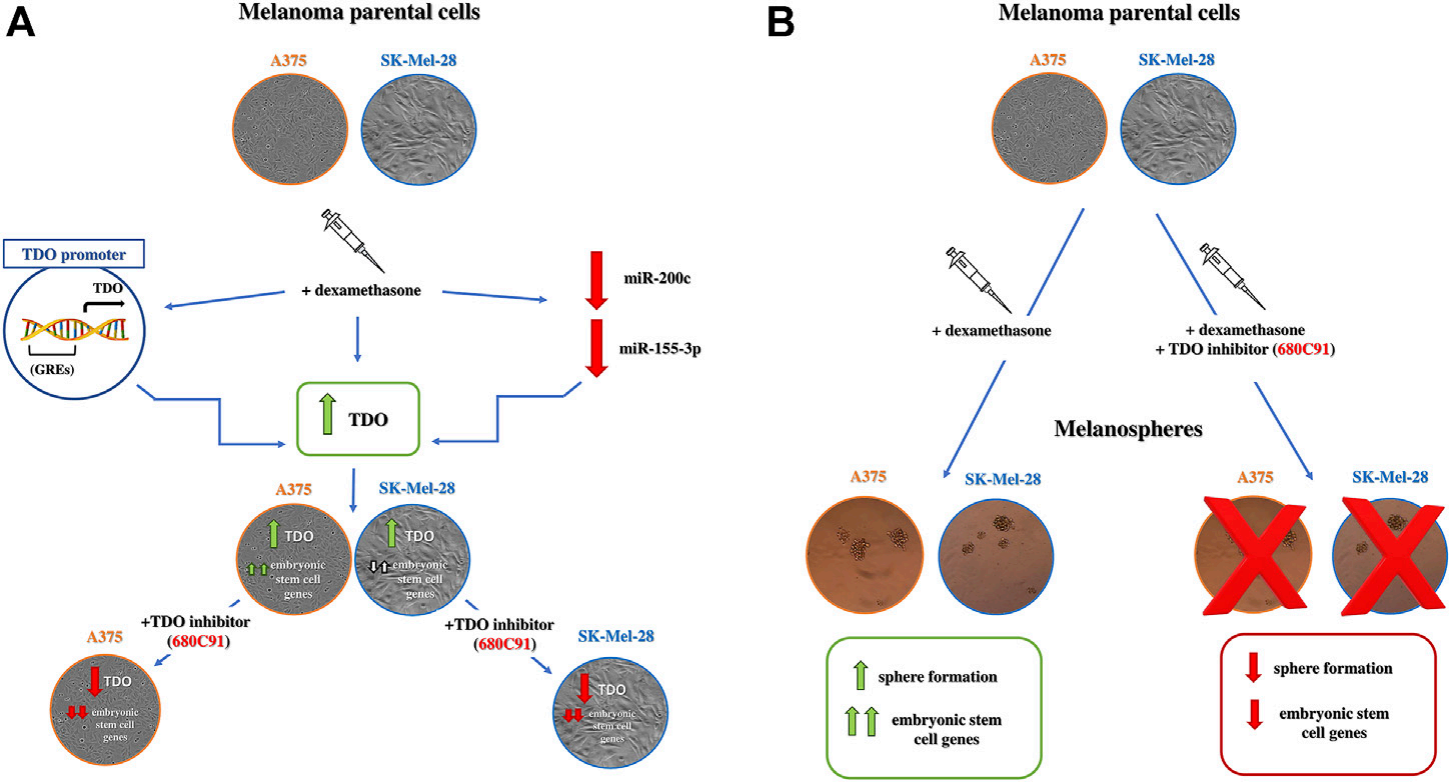
**(A)** Schematic effects of dex on parental melanoma cells. Dex upregulates TDO expression which is essential for embryonic stem cell expression in A375 and with a lesser extent in SK-Mel-28 cells. miR-200-c and miR-115-3p are probably involved in dex-promoted TDO mRNA upregulation. 680C91: selective TDO inhibitor. Green arrows: stimulation; red arrows: inhibition; white arrows: small activation **(B)** Effect of parental cell pretreatment with dex on spherogenesis. Dex strongly stimulates spherogenesis and up-regulation of embryonic stem cell markers in melanospheres through TDO. Green arrows: stimulation; red arrows: inhibition.

Taken together, our results highlight a direct role of TDO in cancer cell property, besides its well-known immune-mediated capacity. As such, targeting TDO could have a double impact on cancer growth inhibition: a direct effect on cancer cells and an indirect one on immune escape. Our data support the notion that TDO’s expression is regulated in an autocrine-paracrine way by dex stimulation. Dex, indeed, upregulates TDO mRNA and protein, the latter required for the acquisition of stem cell features by SK-Mel-28 and A375 cells, that are prevented by 680C91 treatment (Figure 8). Our data are also in line with previous ones demonstrating that in other tumors, TDO-derived Kyn activates AhR in an autocrine/paracrine manner to promote tumorigenesis of brain, breast, and bladder cancer cells (Opitz et al., 2011; Pham et al., 2018; Huang et al., 2020). Thus, inhibiting TDO could be very relevant in melanoma therapy, acting both as immunotherapeutic and chemotherapeutic agent. Therefore, TDO may represent an attractive target, especially when IDO1 does not account for constitutive Trp catabolism (Pilotte et al., 2012) or as a compensatory pathway when IDO1 is pharmacologically impaired.

## Conclusion

Our results provide evidence that TDO may have an important role in melanoma progression. Moreover, dex modulates growth and stemness of melanoma cells, which indeed corroborates clinical evidence suggesting a detrimental effect of systemic steroids during immunotherapy (Indini et al., 2020). Several lines of evidence implicate glucocorticoid receptor signaling in tumorigenesis and cancer progression (Van Der Pompe et al., 1996; Veneris et al., 2017). Thus, targeting GCs pathways to improve cancer immunotherapy success is an attractive strategy.

## Data Availability

The original contributions presented in the study are included in the article/supplementary material, further inquiries can be directed to the corresponding authors.
